# Local control in metastatic neuroblastoma in children over 1 year of age

**DOI:** 10.1186/s12885-015-1082-7

**Published:** 2015-02-21

**Authors:** Maria Antonietta De Ioris, Alessandro Crocoli, Benedetta Contoli, Maria Carmen Garganese, Gianluigi Natali, Paolo Tomà, Alessandro Jenkner, Renata Boldrini, Maria Debora De Pasquale, Giuseppe Maria Milano, Silvia Madafferi, Aurora Castellano, Franco Locatelli, Alessandro Inserra

**Affiliations:** 1 Hematology/Oncology Department, Ospedale Pediatrico Bambino Gesù-IRCCS, Rome, Italy; 2 Surgery Department, Rome, Italy; 3 Imaging Department, Rome, Italy; 4 Pathology Department, Bambino Gesù Children’s Hospital, Rome, Italy; 5 University of Pavia, Pavia, Italy

**Keywords:** Neuroblastoma, Surgery, Metastasis, Radiotherapy, Prognosis

## Abstract

**Background:**

Local control is always considered in metastatic neuroblastoma (NBL). The aim of this study is to evaluate the impact of radical surgery on survival in children over 1 year of age.

**Methods:**

Fifty-eight patients older than 1 year of age with metastatic NBL were treated with conventional plus high-dose chemotherapy with or without addition of local radiotherapy (RT, 21Gy). Surgery was classified as radical surgery (complete resection and gross total resection) or non-radical surgery. The Kaplan-Meier method and the Cox proportional hazard model were used to calculate the probability of progression free and overall survival (PFS and OS) and for multivariate analysis.

**Results:**

The 5-year PFS and OS for patients with radical surgery were 26% (95% CI 14-40%) and 38% (95% CI 23-53%) respectively, while the PFS and OS for patients without radical surgery were 33% (95% CI 10-59%) and 31% (95% CI 10-55%) (respectively, P 0.85 and P 0.42). The 5-year PFS and OS for patients who received RT were 36% (95% CI 19-53%) and 46% (95% CI 26-64%) respectively, while the 5-year PFS and OS for patients who did not receive RT were 22% (95% CI 9-38%) and 27% (95% CI 13-42%) respectively (P 0.02 for PFS). Multivariate analysis confirmed the role of well-known prognostic factors, such as the presence of MYCN amplification, age and response before high-dose chemotherapy.

**Conclusions:**

Our data suggest that the degree of resection does not influence survival in metastatic NBL patients treated with high-dose chemotherapy; local RT contributes to local disease control.

## Background

Neuroblastoma (NBL) is the most common solid extra-cranial tumor of childhood. The clinical course varies from spontaneous tumor regression to an aggressive, poorly responding disease, depending on patient age at diagnosis, metastatic dissemination and MYCN status [1]. Despite intensive treatment, the outcome of high-risk NBL (i.e. metastatic disease or patients with MYCN amplification) remains unsatisfactory: the reported 3-year event-free survival (EFS) is less than 40% in many studies [1-6]. Currently, standard treatment for high-risk NBL is based on intensive systemic chemotherapy, surgery on primary tumor, high-dose chemotherapy followed by autologous bone marrow transplantation (ABMT) and/or peripheral blood stem cell transplantation (PBSC), radiotherapy (RT) on primary tumor bed and differentiating/immunotherapy treatment [1-6]. Local control of NBL, based on surgery and RT on primary site, is considered a valuable option in most international protocols. Nevertheless, the impact of surgery on survival in metastatic NBL treated with an intensive approach remains controversial [7-15]. Some authors reported a favorable outcome in patients who undergo gross total resection (i.e. >95%) of the primary tumor [7-11]; others failed to show an improvement in survival rate after radical surgical excision [12-15]. However, several studies suggested that RT contributes significantly to the prevention of local relapse [16-18].

The aim of this study was to analyze the role of surgery in a series of metastatic NBL in children over 1 year of age diagnosed and treated at the Ospedale Pediatrico Bambino Gesù (OPBG). All patients were treated according to two previously published local protocols based on conventional chemotherapy followed by high-dose chemotherapy [19-21].

## Methods

Children over one year of age with metastatic NBL were classified as having high-risk disease and were enrolled in two consecutive institutional protocols from July 1996 to August 2009. The first protocol was identified as ICE/CECAT [18] and the second as TopoNB [19]. The ICE/CECAT protocol consisted of two courses of ifosfamide/carboplatin/etoposide (ICE), two courses of cyclophosphamide/etoposide/carboplatin/thiotepa (CECAT) or two further ICE courses.

The Topo/NB protocol consisted of two courses of topotecan/cyclophosphamide followed by two courses of ifosfamide/carboplatin/etoposide (ICE) and a later course of cyclophosphamide/doxorubicin/vincristine. The Ethical Committee of Ospedale Pediatico Bambino Gesù IRCCS (ref number 62,10; May, 17th 2010) approved both protocols, as well as this retrospective study. Written informed consent was obtained from the children’s parents or legal guardians. Both protocols were based on conventional induction chemotherapy, surgery on primary tumor and high-dose chemotherapy followed by PBSC rescue and/or ABMT plus 9-cis retinoic acid as previously reported [18-21]. In the second and more recent protocol, the treatment was completed by local RT with 21 Gy on tumor bed before surgery [17].

Surgical resection of primary tumor was performed either at diagnosis or after the 4th or 5 th course of chemotherapy. In detail, patients with tumors considered to be resectable at diagnosis underwent primary surgery, while patients with unresectable tumor at diagnosis and without disease progression after induction chemotherapy underwent delayed surgery.

The same surgical team performed the surgery in all patients. The surgical and pathology reports and imaging before and after surgery, were reviewed for this study. Surgery was classified as “radical” or “not radical”. The “radical” group included patients who had undergone complete resection with no visible tumor or at least a gross total resection (GTR) with less than 5% of visible tumor. The “not radical” group included patients who had undergone a partial resection (PR) with more than 50% tumor volume removal or biopsy only.

### Evaluation of disease

Primary tumor evaluation was done by computed tomography (CT) or magnetic resonance imaging (MRI). Metastatic spread was assessed by total body CT scan and 123-iodine metaiodobenzylguanidine (123I-MIBG) scintigraphy and completed by two bilateral trephines and bone marrow aspirates. Diagnosis and staging were performed according to the International Neuroblastoma Diagnosis and Staging Criteria [22]. Primary tumor response was evaluated using the same investigations as those employed at diagnosis. Responses were assessed according to the International Neuroblastoma Response Criteria [22].

### Statistical analysis

Progression-free survival (PFS) was defined as the time interval from the date of diagnosis to the date of first relapse/progression or the date of the last follow-up for surviving patients. Overall survival (OS) was defined as the time interval between the date of diagnosis and the date of death from any cause or the date of last follow-up for surviving patients. Local progression-free survival (LPFS) was defined as the time interval between the date of diagnosis and the date of first local relapse/progression or the date of the last follow-up. The Kaplan–Meier method was used to estimate survival curves [23], while the log-rank test was used to compare differences between groups. Multivariate analyses were performed using Cox proportional hazards regression model for PFS and OS. All variables with P values >0.2 in univariate analysis were included in the initial model and were then eliminated one at a time in a stepwise fashion to retain only those variables that reached a P value of 0.05 or less in the final models. All P values were 2-sided, with a type-I error rate fixed at 0.05. Variables considered as potentially influencing PFS and OS were: age (either >18 months or <18 months), site of primary tumor, site of metastasis (bone/bone marrow/lymph node or lymph node alone or others), MYCN status (MYCN amplified versus MYCN non amplified or MYCN gain), induction regimen, response before surgery, RT, response at the end of induction and quality of surgery, defined as radical or non-radical surgery. Analyses were performed using the Stata 9.0 statistical software package (StataCorp LP, TX, USA).

## Results

This study evaluated 58 children over one year of age diagnosed with metastatic NBL at the OPBG and enrolled into two local treatment protocols. Patient characteristics are summarized in Table 1. Median age at diagnosis was 36 months (range 13–216), 11 of the 58 patients (19%) being younger than 18 months. Surgery was performed at diagnosis in 7 patients and was radical in 6 of them. In 47 (81%) patients tumor resection was performed after induction chemotherapy and was complete in 39 (83%). MYCN status was available for 56 patients; out of 43 patients with radical surgery, 13 presented MYCN amplification while 6 patients out of 13 without radical surgery had MYCN amplification (P 0.29).

**Table 1:** **Patient characteristics**

			*N*	%
**Age**	Median (months)	36		
	Range (months)	13-216		
**Gender**	Male		35	60
	Female		23	40
**Site of Metastasis**	Bone/Bone Marrow/Lymph node		48	83
	Lymph node alone		3	5
	Others		7	12
**Primary Sites**	Retroperitoneal		12	21
	Adrenal Gland		41	71
	Thorax		5	8
**MYCN**	Amplified		19	34
**(available for 56 pt)**	Non amplified or MYCN gain		37	66
**Induction CT**	ICE/CECAT		21	36
	Topo NB		37	64
Primary Tumor Reduction before Surgery*	<50%		4	8
	>50%, <95%		34	68
	>95%		12	24
**Surgery**	Radical		45	78
	Not Radical		13	22
Response before HDC**	CR/VGPR		21	37
	PD		11	20
	PR		24	43
**RT**	Yes		28	48
	No		30	52

LEGEND: pt, patient; BX, biopsy; CR, complete remission; VGPR, very good partial remission; PD, progressive disease; PR, partial response. For “others” site of metastasis was considered Bone or/and Bone Marrow or/and Lymph node plus lung or/and liver metastatis.

*In 7 patients surgery was performed at diagnosis, one patient died for an acute bleeding after accidental removal of central vein catheter.

**Two patients were not considered for this analysis; one patient died for acute bleeding and the later one died for acute renal failure.

In this series, there was no intraoperative death; one patient died after surgery due to acute renal failure. Nephrectomy was performed in 7 patients (13%), polar nephrectomy for renal NBL and partial liver removal in one patient each. Post-surgery abscess, massive blood loss (defined as loss of one blood volume in 24 hours, or 50% loss of one blood volume in 3 hours, or losses over 1,45 ml/kg/min for 20 minutes, or transfusion of over > 40 ml/kg of red cells) [24,25], or the need for mechanical ventilation for more than 5 days were not recorded.

### Survival

**Figure 1:**
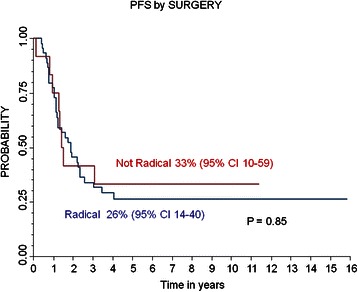
**Progression Free Survival (PFS) and Overall Survival (OS) of the whole population.**

**Figure 2:**
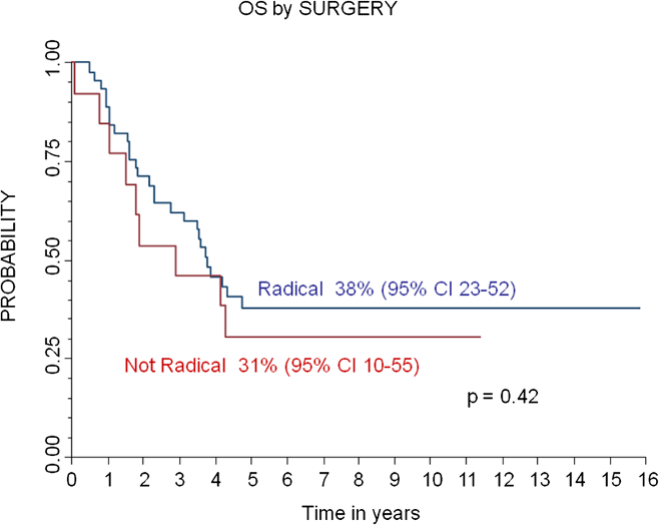
**Local Progression Free Survival (PFS) and PFS of whole population and by surgery; Overall Survival (OS) by surgery.**

The median follow-up for the entire cohort was 45 months (range 1 month-16 years). The 5-year PFS and OS were 28% (95% CI 17-40%) and 36% (95% CI 24-49%), respectively, while the 5-year LPFS was 72% (CI 25-83%). Out of 58 patients, 41 (71%) died at a median time from diagnosis of 28 months (range 1–94 months): 39 died due to relapsed/resistant disease and two from complications (the patient who died of renal failure after surgery and one who died due to acute bleeding after accidental removal of the central venous catheter). Relapse/progression occurred in 39/58 (67%) patients after a median time from diagnosis of 15 months (range 6–49 months). Local relapse occurred in 12/58 (20%) patients; none of them had received RT, while radical surgery had been performed in 10 of the 12 patients. Local relapse was observed in 7 out of 19 (37%) patients with MYCN-amplified tumor, while relapses were recorded in only 5 out of 37 (14%) patients with non-MYCN-amplified tumors (*P* < 0.005). The 5-year PFS and OS for patients with radical surgery were 26% (95% CI 14-40%) and 38% (95% CI 23-53%) respectively, while the PFS and OS for patients without radical surgery were 33% (95% CI 10-59%) and 31% (95% CI 10-55%) (P 0.85 for PFS and P 0.42 for OS, (Figures 1 and 2). The 5-year PFS and OS for patients who did or did not receive RT were 36 % (95% CI 19-53%) and 46% (95% CI 26-64%) respectively, and 22% (95% CI 9-38%) and 27% (95% CI 13-43%) respectively (*P* < 0.02 for PFS and *P* = 0.23 for OS).

On univariate analysis, age, site of metastasis, MYCN status, response before high-dose chemotherapy and RT were factors influencing patient outcome (See Table 2 for more details). The final model of the multivariate analysis showed age, MYCN status and response before high-dose chemotherapy to be prognostic factors for PFS and OS. MYCN amplification was associated with poor OS (HR 2.24, P = 0.043) as 183 were age >18 months (HR 4.52, *P* = 0.042) and Progressive Disease (PD) at the end of induction (HR 5.75, P < 0.001). Moreover, in this series RT was a protective factor for OS; patients who did not receive RT presented an HR of 2.36 (*P* = 0.025) for OS. Response before high-dose chemotherapy was shown to be the major prognostic factor for PFS; patients with PR presented an HR of 3.15, *P* = 0.006. Regarding LPFS, response before high-dose chemotherapy, RT and MYCN status were found to be the major prognostic factors. The 5-year LPFS in patients who did or did not receive RT was 100% and 48% (95% CI 27-67%) respectively (P <0.001) (see also Table 3 for more details). In the final model of multivariate analysis, MYCN amplification was shown to be a prognostic factor for local recurrence with an HR of 2.16 and *P* = 0.043.

**Table 2:** **Univariate analysis of outcome’s predictive factors**

		Pts	PFS 5 years	95% CI	Univariate analysis	OS 5 years	95% CI	Univariate analysis
			%		*P*value	%		*P*value
**Age**	12-18 months	11	64	30-85	**0.01**	73	37-90	**0.03**
	>18 months	47	19	9-32		28	16-41	
**Site of Metastasis**	Bone/Bone Marrow/Lymph-node	48	25	14-39	**0.03**	35	22-49	**0.03**
	Lymph-node alone	3	100	-		100	-	
	Others	7	14	1-46		14	1-46	
**Primary Sites**	Retroperitoneal	12	17	3-41	0.28	11	1-38	0.21
	Adrenal Gland	41	28	15-42		40	25-55	
	Thorax	5	60	13-88		60	13-88	
**MYCN**	Amplified	19	30	11-52	0.24	26	10-47	**0.00**
**(available for 56 pt)**	Non amplified or MYCN gain	37	29	16-44		44	27-59	
**Induction CT**	ICE/CECAT	21	20	6-39	0.59	29	12-48	0.19
	Topo NB	37	33	19-49		40	24-56	
Primary Tumor Reduction before Surgery*	<50%	35	22	9-38	0.52	38	21-54	0.43
	>50%, <90%	4	0	-		0	-	
	>90%	12	33	10-59		33	10-59	
**Surgery**	Radical	45	26	14-40	0.85	38	23-52	0.42
	Not Radical	13	33	10-59		31	10-55	
Response before HDC**	CR/VGPR	21	62	38-79	**<0.001**	65	40-82	**<0.001**
	PD	11	0	-		10	1-33	
	PR	24	13	3-29		29	13-48	
**RT**	Yes	28	36	19-53	0.23	46	26-64	**0.02**
	No	30	22	9-38		27	13-43	

LEGEND: pt, patient; BX, biopsy; CR, complete remission; VGPR, very good partial remission; PD, progressive disease; PR, partial response.

*In 7 patients surgery was performed at diagnosis, one patient died for an acute bleeding after accidental removal of central vein catheter.

**Two patients were not considered for this analysis; one patient died for acute bleeding and the later one died for acute renal failure.

Progression-Free Survival and Overall Survival according to predictive factors.

**Table 3:** **Univariate analysis of factors predictive for Local PFS**

		Pts	Local PFS	95% CI	Univariate analysis
			%		*P*value
**Age**	12-18 months	11	100	-	**0.04**
	>18 months	47	63	42-76	
**Site of Metastasis**	Bone /Bone Marrow/ Lymph-node	48	75	56-86	**0.07**
	Lymph-node alone	3	100	-	
	Others	7	27	1-69	
**Primary Sites**	Retroperitoneal	12	59	16-86	0.50
	Adrenal Gland	41	76	55-88	
	Thorax	5	60	13-88	
**MYCN**	Amplified	19	51	23-74	**0.00**
**(available for 56 pt)**	Non-amplified or MYCN gain	37	80	59-91	
**Inducion CT**	ICE/CECAT	21	55	28-76	0.12
	Topo NB	37	83	53-93	
Primary Tumor Reduction before Surgery*	<50%	35	78	54-90	**0.04**
	>50%, <90%	4	50	1-91	
	>90%	12	47	17-72	
**Surgery**	Radical	45	68	49-81	0.35
	Not Radical	13	90	47-99	
Response before HDC**	CR/VGPR	21	86	62-95	**<0.001**
	PD	11	0	-	
	PR	24	64	34-83	
**RT**	Yes	28	100	-	**<0.001**
	No	30	48	27-67	

LEGEND: pt, patient; BX, biopsy; CR, complete remission; VGPR, very good partial remission; PD, progressive disease; PR, partial response.

*In 7 patients surgery was performed at diagnosis, one patient died for an acute bleeding after accidental removal of central vein catheter.

**Two patients were not considered for this analysis; one patient died for acute bleeding and the later one died for acute renal failure.

## Discussion

Treatment of metastatic NBL in children over 1 year of age continues to represent a challenge for pediatric oncologists. Local control is always considered in the therapeutic strategy for children with metastatic NBL; however, its role in the treatment of patients receiving high-dose chemotherapy remains a subject of medical debate [26]. In this series of homogeneously treated metastatic NBL, the quality of surgery had no impact on survival. Indeed, the PFS and OS of patients who underwent radical surgery were comparable with those of patients with partial removal of primary tumor or biopsy only. Multivariate analysis showed that previously identified prognostic factors, i.e. MYCN amplification, age at diagnosis and response at the end of the induction phase, represented the major prognostic factors in the patient cohort.

This is the first single-center report on the impact of local control in terms of survival in patients treated with high-dose chemotherapy. Previously published data reported on patients enrolled in studies - mostly multi-center studies - with randomization between high-dose chemotherapy and conventional therapy, or with different post-induction treatments [7-15].

There are still conflicting reports in the literature concerning the role of radical surgery [26]. Some Authors reported the absence of any advantage in terms of survival probability for patients with CR of the primary tumor, as it is almost impossible to achieve a complete absence of microscopic residual disease on the tumor bed [27]. However, in a single center study analyzing cases diagnosed over more than 20 years, LaQuaglia et al. noted that gross tumor resection correlated with an improved outcome in terms of both local control and OS [8]. In a single center study, McGregor et al. observed an advantage for gross total resection in terms of survival [11]. In both these series [8,11], patients received different post-induction treatments.

Adkins et al. [9] showed a trend toward improved survival for complete resection in high risk NBL treated according to the CCG-3891 study. This study included metastatic patients over 1 year of age and patients with localized MYCN-amplified tumor. The randomized CCG-3891 study compared high-dose chemotherapy followed by ABMT with maintenance chemotherapy [3]. Recently, Simon et al. [15] reported no impact of primary tumor surgery on local control and survival in 278 cases of metastatic NBL diagnosed in children over 18 months of age, enrolled in the German clinical trial NB97 and with no progression, relapse or death during the first 120 days of induction chemotherapy. This study was a randomized trial comparing high-dose chemotherapy followed by ABMT with oral maintenance chemotherapy [4,14].

In this paper, the authors discuss whether a single-center study or a multi-center study is the best setting for evaluating the impact of surgery in metastatic NBL. Probably, the single center series is more suitable for this analysis since, in such studies, all the procedures are performed by the same highly trained surgeons, thus avoiding a “different degrees of surgeons’ expertise” bias. Although the number of patients enrolled in our study was limited, the data clearly suggest that the type of surgery has no impact in terms of survival in metastatic NBL in children over one year of age treated with high-dose chemotherapy.

In this series, primary surgery was performed in about 10% of patients, achieving in most cases a CR of primary tumor, whereas, after the induction phase, surgery was performed in more than 90% of patients. Overall, radical surgery was performed in 80% of patients. Nephrectomy – recorded in 13% of patients - was the major complication. The International Neuroblastoma Risk Group modified the criteria for classification of localized NBL, including the image-defined risk factors (IDRFs), which assess both the staging of the tumor and the criteria for identifying and predicting the surgical risks for vital structures [27,28]. IDRFs could be a useful tool for the surgeon, even for patients with metastatic high-risk NBL, in that they predict and possibly help to avoid both acute and late surgical complications such as renal failure after nephrectomy, renal atrophy after adrenal resection, and ejaculatory dysfunction secondary to pelvic resection. In this series, the same surgical team performed both the pre-surgical patient evaluation and the surgical procedure. The involvement of a highly-experienced surgical team will probably result in a lower rate of post-operative complications, even in patients with several surgical risk factors. As underlined by Simon [15], surgery should be conservative and the risk of removing the kidney or any other vital organ should be carefully weighed against any potential benefits.

As previously reported, RT after surgery seems the best option to control local relapse [16-18]. Our findings confirm these previously published data since, in this cohort, RT was associated with a lower local recurrence rate. Indeed, the LPFS in patients who received RT was 100% and contributed to improving the probability of OS considering that patients who did not receive RT had an HR of 2.36 for OS (*P* = 0.025).

## Conclusions

In conclusion, we show that the extent of surgery had no impact on survival in a our single center series of homogeneously treated metastatic NBL. Surgery may help to achieve the best disease control before high-dose chemotherapy and should be proposed to all patients after the induction phase; in centers with experienced surgeons, it could be discussed at diagnosis after considering the IDRFs.
